# Stomatitis Materni

**Published:** 1859-08

**Authors:** David Prince

**Affiliations:** Jacksonville, Ill.


(From The Chicago Medical Journal.)

### STOMATITIS MATERNI.

BY DAVID PRINCE, M. D.

DR. BRAINARD: *Dear Sir*,—I have been much interested in reading the article on Nursing Sore Mouth, by Dr. T. J. W. Pray, of Dover, N. H., published in the May number of the *Chicago Medical Journal.* Allow me to contribute a short note as mere hints or suggestions.

Dr. Pray thinks the disease to be aphthæ, and says (page 307): "We regard this disease as local in its nature,"

Turning to Hooper's Medical Dictionary, which happened to be lying upon my table, I find aphthæ defined as a disease which appears in small white ulcers upon the tongue, gums, and around the mouth and palate, resembling small particles of curdled milk. Turning to Watson's Practice (page 482 American edition, 1847): "Aphthæ consists in small, irre[illegible] lar, but usually roundish, white specks or batches, scat[illegible] over the tongue and lining membrane of the mouth and [illegible] lips," etc. "They look like little drops of tallow or [illegible] of curd sprinkled over those parts; they project above the surrounding surfaces, and, in fact, they are formed by elevated portions of the mucous epidermis, [illegible] ing a small quantity of sordes or gelatinous fluid whic[illegible] rates epidermis from the subjacent corium." This port[illegible] is very distinct and true to life. Any one who has [illegible]

sore mouth in an infant must recognise the truth of the description at once. In this region the women usually call the disease *canker.*

Nursing sore mouth is endemic in and around Jacksonville, and I have frequently seen and treated it: but I have never seen it as aphthæ, according to the definition of Hooper and Watson, just quotod. As it has fallen under my observation, the predominant characteristic is *ulceration.* Any occurence of vesicles is so insignificant as not to attract attention. The general surface of the tongue and mouth is a little darker than usual, and the woman who has ever before had the disease, recognises its approach before any ulceration can be discovered. When the disease proceeds unchecked, these ulcers assumes various forms,—sound, ragged and cracked, or fissured. There is never a white crust which is made a part of the definition of aphthæ. The feeble are more often afflicted, but no condition of health seems to produce an exemption.

The treatment in this region among the physicians with whom I am acquainted, has come to be a settled and sure thing. It is very rarely the case that a nursing mother is required to wean her child on account of this affection, unless it has become obstinate by neglect, and the general health reduced by its irritation, and the attendant inability to take nourishment.

The favorite remedy is iodide of iron. The liquor ferri iodidi of the U. S. P. is combined with an equal quantity of compound syrup of sarsaparilla, for the more agreeable taste, and of this a common sized teaspoonful is directed to be taken three times a day. In nine cases out of ten this cures, whether before or after delivery. (The disease is an attendant of pregnancy as well as of lactation.)

The next remedy in favor is the chlorate of potash. A saturated solution is made, and it is used both as a local application and as an internal remedy. A tablespoonful of the solution may be held in the mouth for a few second and then swallowed, or if only a local application is intended, it can be thrown out of the mouth. This may be done from three to twelves times a day.

These two remedies may be used singly or in combination.

To make the matter short the disease does *not* correspond with the definition of aphthæ, and therefore should not be called by that name; and it cannot be a mere *local* disease, or it would not be so readily controlled by an internal remedy, as in the use of the iodide of iron.

Respectfully Yours, DAVID PRINCE.

JACKSONVILLE, ILL., May 24th, 1859.

